# Vasostatin-1: A novel circulating biomarker for ileal and pancreatic neuroendocrine neoplasms

**DOI:** 10.1371/journal.pone.0196858

**Published:** 2018-05-03

**Authors:** Andrea Corsello, Luigi Di Filippo, Sara Massironi, Federica Sileo, Anna Dolcetta Capuzzo, Marco Gemma, Claudia Carlucci, Claudio Cusini, Barbara Colombo, Alice Dallatomasina, Giulia Maria Franchi, Angelo Corti, Marco Federico Manzoni

**Affiliations:** 1 Department of General Medicine and Endocrine Tumor Unit, San Raffaele Scientific Institute, Milan, Italy; 2 Department of Gastroenterology and Endoscopy Unit, Fondazione IRCCS Ca' Granda Ospedale Maggiore Policlinico, Milan, Italy; 3 Department of Neurointensive Care, San Raffaele Scientific Institute, Milan, Italy; 4 Clinical study & Data Management Unit, Haematology Project Foundation, Vicenza, Italy; 5 Division of Experimental Oncology, San Raffaele Scientific Institute, Milan, Italy; 6 San Raffaele Vita-Salute University, Milan, Italy; UPR 3212 CNRS -Université de Strasbourg, FRANCE

## Abstract

**Background:**

Chromogranin A (CgA) is a plasma biomarker widely used in the follow-up of patients with neuroendocrine neoplasms (NENs). However, its accuracy as a tumor biomarker is relatively low because plasma CgA can increase also in patients with other diseases or in subjects treated with proton-pump inhibitors (PPIs), a class of widely-used drugs.

**Methods:**

In the attempt to identify a more reliable biomarker for NENs, we investigated, by ELISA, the circulating levels of full-length CgA (CgA_1-439_) and of various CgA-derived fragments in 17 patients with ileal or pancreatic NENs, 10 healthy controls, and 21 healthy volunteers before and after treatment with PPIs.

**Results:**

Patients with ileal or pancreatic NENs showed increased plasma levels of total-CgA and CgA_1-76_ fragment (vasostatin-1, VS-1) compared to controls [median (25^th^-75^th^-percentiles); total-CgA: 1.85 nM (1.01–4.28) *vs* 0.75 nM (0.52–0.89), *p* = 0.004; VS-1: 2.76 nM (1.09–7.10) *vs* 0.29 nM (0.26–0.32), *p*<0.001, respectively], but not of CgA_1-439_ or CgA_1-373_ fragment. VS-1 positively correlated with total-CgA (r = 0.65, *p*<0.001). The Receiver Operating Characteristic area under the curve was 0.9935 for VS-1 and 0.8824 for total-CgA (*p* = 0.067). Treatment of patients with somatostatin analogues decreased both total-CgA and VS-1. In contrast, administration of PPIs increased the plasma levels of total-CgA, but not of VS-1.

**Conclusion:**

These findings suggest that plasma VS-1 is a novel biomarker for ileal and pancreatic NENs. Considering that VS-1 is a well-defined fragment not induced by proton-pump inhibitors, this polypeptide might represent a biomarker for NENs diagnosis and follow-up more accurate and easier to standardize than CgA.

## Introduction

Human Chromogranin A (CgA), a 439-residue-long protein present in the secretory granules of many normal and neoplastic neuroendocrine cells, currently represents the main biomarker for neuroendocrine neoplasms (NENs) [1, 2]. CgA is exocytotically released in circulation, to reach approximately 0.5 nM levels in healthy subjects and up to 100–500–fold higher values in NENs patients [3–5]. Elevated levels of circulating CgA have been reported also for sub-populations of patients with non-small-cell lung cancer, prostate or breast cancer, or for patients with heart failure, renal failure, hypertension, rheumatoid arthritis, atrophic gastritis, liver disease, inflammatory bowel disease, sepsis and other inflammatory diseases [4–13]. Elevated levels of circulating CgA are present also in subjects treated with proton-pump inhibitors (PPIs), a class of drugs largely used in patients [14, 15]. Therefore, although plasma CgA is still widely used as a biomarker for NENs, its clinical utility is limited to prognostic stratification of patients with advanced disease [16, 17].

A further complication for the use of CgA as tumor biomarker is that CgA assays are difficult to standardize because this protein is a heterogeneous analyte due to extensive proteolytic processing and differential post-translational modifications [18–22]. The proteolytic processing of CgA can be triggered by intra-granular and/or extracellular proteases including prohormone convertase 1 and 2, furin, cathepsin L, plasmin and thrombin [23]. CgA, upon proteolysis, can give rise to several biologically active peptides, such as vasostatin-1 (VS-1, human CgA_1-76_), catestatin (human CgA_352-372_), pancreastatin (human CgA_250-301_), serpinin (CgA_411-436_) and other larger polypeptides consisting of CgA molecules lacking part or most of the C-terminal region (e.g. CgA_1-373_) [10, 24–27]. These peptides have been implicated in the regulation of vascular tension, angiogenesis, endothelial-barrier function, cardiovascular function, inflammation, gastrointestinal motility and glucose and calcium metabolism [3]. Regarding angiogenesis, it has been recently proposed that CgA and its fragments can form a balance of pro- and anti-angiogenic factors. For example, while the full-length CgA (hereinafter CgA_1-439_) and VS-1 can inhibit angiogenesis, the CgA_1-373_ fragment can promote angiogenesis [20, 28].

In the attempt to identify a more reliable biomarker for NENs we investigated the circulating levels of CgA_1-439_ and of the anti- and pro-angiogenic fragments VS-1 and CgA_1-373_ in patients affected with ileal or pancreatic NENs, before and after therapy with somatostatin analogues (SSAs), and in healthy volunteers, before and after administration of PPIs. Plasma levels of each polypeptide were measured using specific ELISAs. Furthermore, considering that most assays typically used for CgA detection can detect mixtures of full-length CgA and fragments [23], we also used an ELISA with a broader specificity, based on antibodies capable of detecting full-length CgA and fragments lacking the C-terminal region and larger than VS-1 (here defined as “total-CgA”), thus unable to detect VS-1.

## Materials and methods

### Plasma samples collection

We evaluated blood samples from 17 patients with pancreatic or ileal NENs diagnosed between 1996 and 2012 at IRCCS Ca' Granda Ospedale Maggiore Policlinico, Milan, Italy. We included only patients with G1/G2 neoplasms since G3 neoplasms are frequently poorly differentiated and therefore may lose the ability to secrete CgA [29, 30]. Blood samples were collected at diagnosis and at follow-up visit after administration of Octreotide LAR.

Blood samples were also collected from 10 healthy donors and 21 healthy volunteers before and after therapy with oral pantoprazole (40 mg/day, 14 days). Of the 21 healthy volunteers, 16 (76.2%) were females and 5 (23.8%) were males. 19 subjects were Caucasians (90.5%) and 2 subjects (9.5%) were Hispanics. Median age was 52.0 years.

Blood was collected in EDTA-containing tubes and plasma was obtained immediately by centrifuging each sample (2000g for 15 min, room temperature). The plasma samples were then stored at < -20°C until assay.

### Data collection

Diagnosis of NENs was confirmed by conventional histology and immunohistochemical studies (CgA, synaptophysin, neuron-specific enolase).

All patients were classified according to primary tumor sites (ileus or pancreas), and WHO-2010 Classification [31].

All the 31 healthy subjects underwent a brief interview in order to collect demographic data and relevant clinical information and to enlist only subjects without comorbidities that might affect CgA plasma levels. We excluded subjects with kidney disease (Glomerular Filtration Rate < 60 ml/min), history of neoplasia, liver failure (Child-Pugh score > 4), inflammatory diseases (inflammatory bowel disease, rheumatoid arthritis), chronic atrophic gastritis, PPIs therapy in the last 30 days or uncontrolled hypertension (BP > 140/90 mmHg). Written informed consent was obtained from all participants and the study was approved by the San Raffaele Ethics Committee (NTBK001).

### Reagents

Mouse monoclonal antibodies (mAbs) 5A8 and B4E11 (epitope: CgA_53-57_ and CgA_68-71_, respectively), rabbit polyclonal antisera αVS-I(76), αCgA(373), αCgA(439), raised against the peptide CgA_71-76_, CgA_369-373_, and CgA_435-439_, respectively and rabbit polyclonal antiserum αCgA(FRs), raised against recombinant human CgA, were produced and characterized as described previously[19, 32, 33].

### Sandwich ELISAs

**Fig 1:**
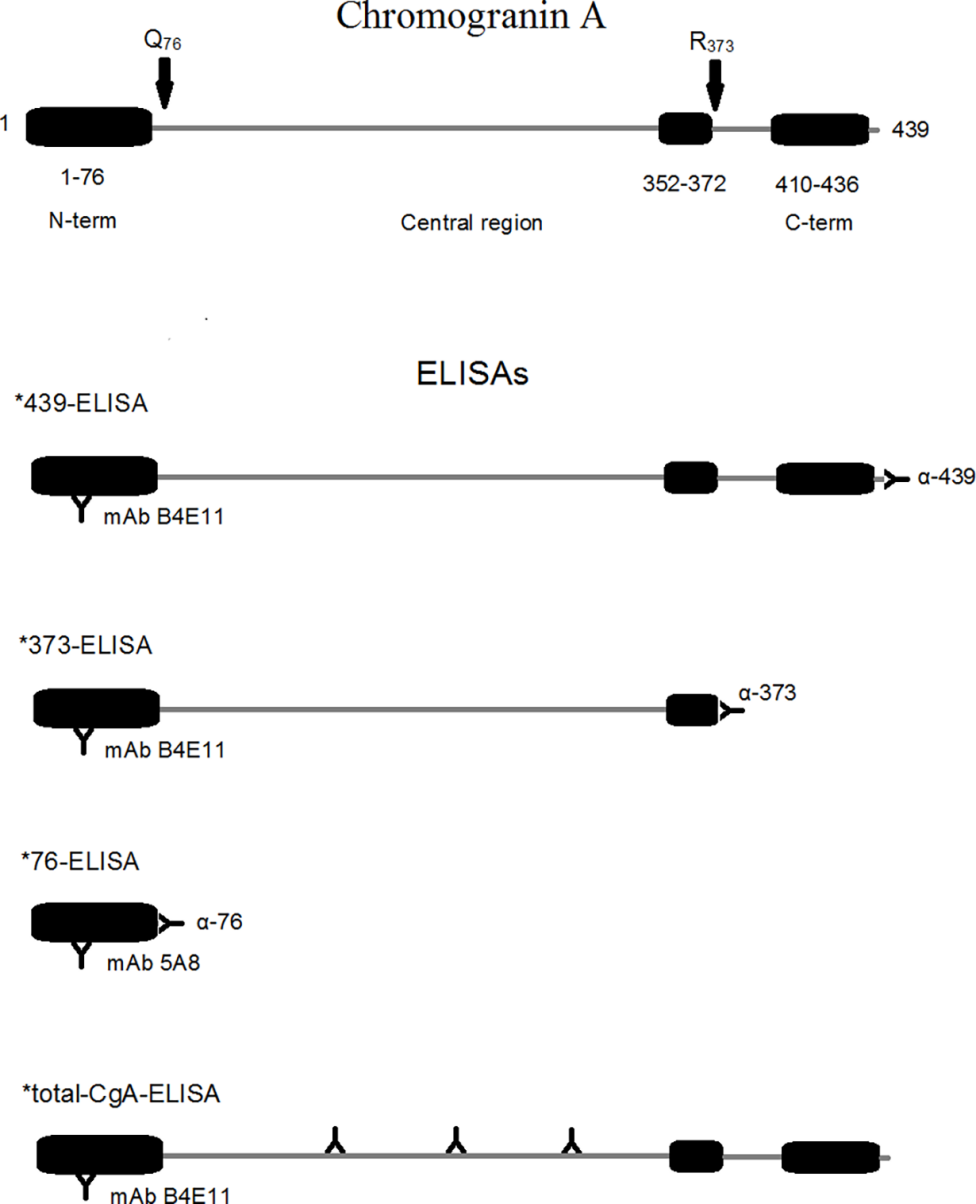
Schematic representation of the ELISAs used to detect CgA and its fragments. Upper panel—Schematic representation of human full-length CgA (439-residues long, CgA_1-439_): Q_76_ and R_373_ cleavage sites are indicated; vasostatin-1 (CgA_1-76_, VS-1), catestatin (CgA_352-372_) and serpinin regions (CgA _410–436_) are boxed. Lower panel—Schematic representation of the *439-, *373-, *76-, and *total-CgA-ELISAs (sandwich ELISA). The epitope location of mAb B4E11 and 5A8 (capturing antibodies), and α439, α373, α76 and αFRs (detecting antibodies) is shown. The rational for using different capturing antibodies (mAb B4E11 and mAb 5A8) in the different assays relies on the fact that, in the case of *76-ELISA, mAb B4E11 and α76 cannot form molecular sandwiches, because of steric hindrance. *439-ELISA can detect molecules with N-terminal domain and intact C-terminal region (i.e. “full-length CgA”), but not fragments; *373-ELISA can selectively detect CgA_1-373_, but not CgA_1-439_; *76-ELISA can selectively detect CgA_1-76_, but not the other fragments; total-CgA-ELISA can detect full-length CgA plus fragments containing the N-terminal and all or part of the central and C-terminal regions (FRs) including CgA_1-373,_ but not CgA_1-76_.

CgA and CgA fragments in plasma samples were detected using four sandwich ELISAs, called *76, *373, *439, and total-CgA ELISA (see Fig 1 for a schematic representation of each assay and the antibodies used) previously described [19].

Assay validation experiments [19, 20] showed that these ELISAs can selectively detect: (1) the N-terminal fragment CgA_1-76_ (VS-1) (*76−ELISA); (2) the fragment CgA_1-373_ (*373–ELISA); (3) full-length CgA (*439–ELISA); (4) full-length CgA plus fragments containing the N-terminal and all or part of the central and C-terminal regions, including CgA_1-373_ (hereinafter called “total-CgA”−ELISA). Of note, this assay cannot detect CgA_1-76_. Intra- and inter-assay coefficient of variation (CV), limits of detection and quantification and specificity of each ELISA are shown in S1 Table.

### Statistical analysis

Statistical analysis was performed using Stata, version 11.1 (StataCorp, College Station, TX, USA). Normality of distribution of analyzed variables was evaluated with visual inspection, Shapiro-Wilk normality test, and Skewness-Kurtosis test. Continuous variables were reported as mean and SD if normally distributed, otherwise they were reported as median and interquartile range (IQR). A *p*<0.05 was considered significant in all tests performed. In case-control analysis, CgA variables and rates were not normally distributed and were compared with the Wilcoxon rank-sum (Mann-Whitney) test. Descriptive data (median, IQR, min, max) were reported in the original units of measure or as true rates.

Since variables and rates were log-normally distributed, a parallel analysis with correction for age could be run, in which variables were previously transformed into their logarithm (± a constant minimizing skewness) and analyzed with analysis of covariance (ANCOVA).

Differences between post-SSAs therapy and pre-SSAs therapy variables (“deltas”) were not normally distributed and were compared with the Wilcoxon signed-rank test. No convenient transformation of the “delta” variables to make them normally distributed was found.

## Results

### Sample description

The characteristics of patients and the classification according to WHO-2010 are listed in Table 1. Of note, none of the patients was taking PPIs.

**Table 1:** Sample description (NENs patient characteristics).

	**Site of Origin**		**Total**
	*Ileus*	*Pancreas*	
**Age: mean±SD, (years)**	60.95±12.17	61.74±14.12	61.28±12.58
	n (%)	n (%)	n (%)
**Sex**			
Male	5 (50)	0 (0)	5 (29.4)
Female	5 (50)	7 (100)	12 (70.6)
Total	10 (100)	7 (100)	17 (100)
**WHO-2010**			
Grade 1	8 (80)	3 (42.8)	11 (64.7)
Grade 2	2 (20)	4 (57.2)	6 (35.3)
Total	10 (100)	7 (100)	17 (100)
**Functioning NENs**			
Carcinoid syndrome	9 (90)	0 (0)	9 (52.9)
Verner-Morrison	0 (0)	1 (14.3)	1 (5.9)
**Non-functioning NENs**	1 (10)	6 (85.7)	7 (41.2)
Total	10 (100)	7 (100)	17 (100)
**Comorbidity**			
MEN-1^a)^	0 (0)	0 (0)	0 (0)
Hypertension	5 (50)	3 (42.8)	8 (47)
Atrophic Gastritis	0 (0)	0 (0)	0 (0)
Liver Failure	1 (10)	0 (0)	1 (5.9)
Other neoplasms	0 (0)	0 (0)	0 (0)
Chronic Kidney Disease	0 (0)	0 (0%)	0 (0%)
**No comorbidity**	4 (40)	4 (57.2)	8 (47.1)
Total	10 (100)	7 (100)	17 (100)
**Treatment**			
Octreotide LAR^b)^	9 (90)	5 (71.4)	14 (82.3)

a) Multiple Endocrine Neoplasia type 1

b) Somatostatin analogues (SSAs)

### The circulating levels of total-CgA and VS-1 are increased in patients with ileal or pancreatic NENs

**Fig 2:**
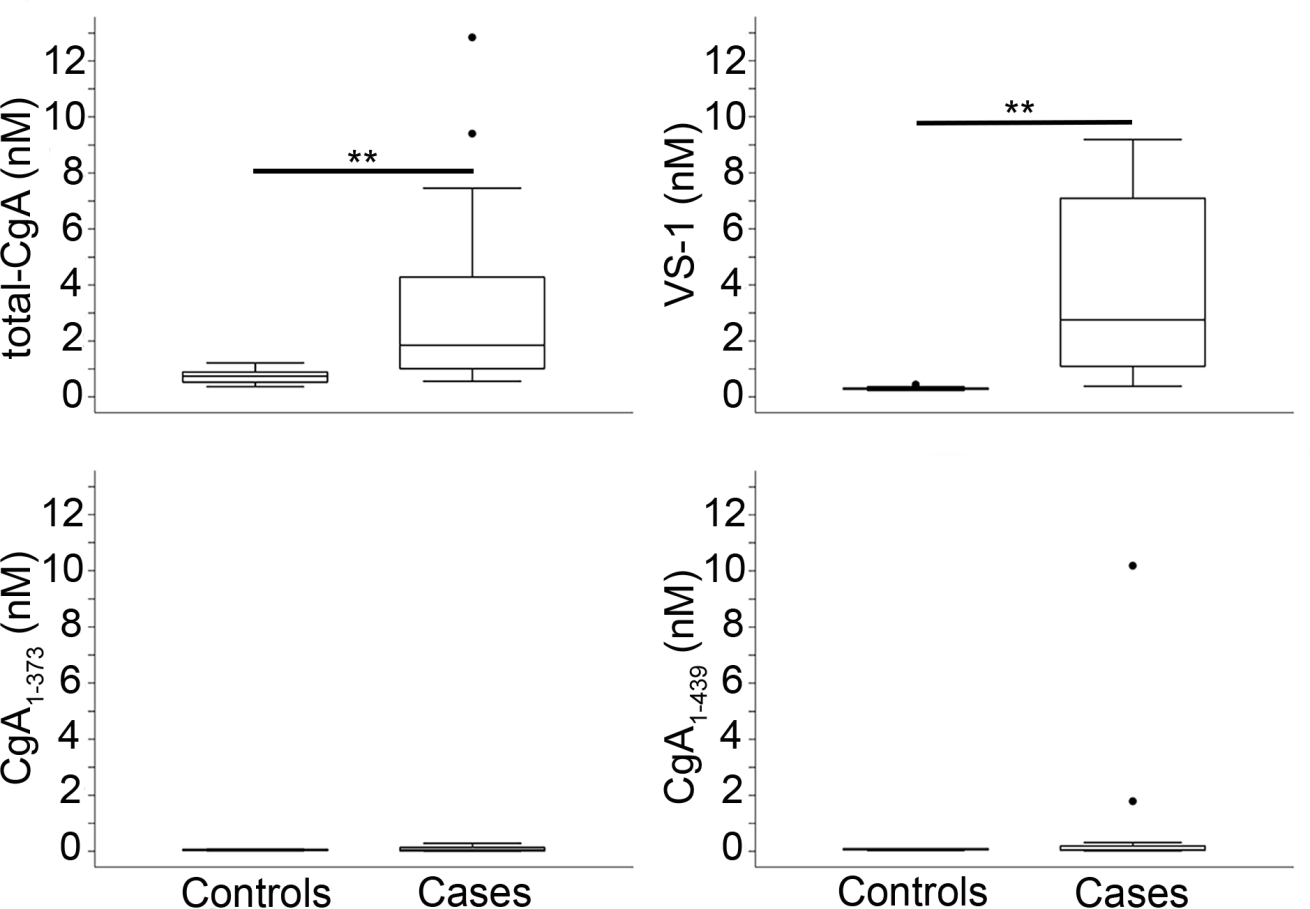
Plasma levels of CgA and its fragments in patients with ileal and pancreatic NENs (cases) and healthy subjects (controls). Box-plots with median (middle line), 75^th^ percentile (top line) and 25^th^ percentile (bottom line). The top and bottom whiskers represent the upper and lower adjacent values, respectively. Values outside the whiskers are plotted individually (circles). Cases (n = 17); controls (n = 10). **(*p* < 0.01), by analysis of covariance.

The plasma samples of 17 patients with ileal or pancreatic NENs and 10 healthy subjects (controls) were analyzed using ELISAs specific for CgA_1-439_, VS-1, and CgA_1-373_, and an ELISA capable of detecting mixtures of full-length CgA and fragments lacking the C-terminal region but unable to detect VS-1 (total-CgA) [19, 20]. The results (Fig 2) show that the median values of total-CgA and VS-1 were increased in patients compared to controls [median (25^th^-75^th^ percentiles), total CgA: 1.848 nM (1.010–4.276) vs 0.752 nM (0.522–0.899), *p* = 0.004; VS-1: 2.757 nM (1.093–7.098) vs 0.288 nM (0.263–0.315), *p*<0.001, respectively]. In contrast, no difference was found regarding CgA_1-439_ and CgA_1-373_. Of note, the fold-increase of VS-1 in patients was markedly higher than that of total-CgA (9.5– *vs* 2.4–fold, respectively).

### The N-terminal proteolytic processing of CgA is increased in patients with ileal or pancreatic NENs

**Fig 3:**
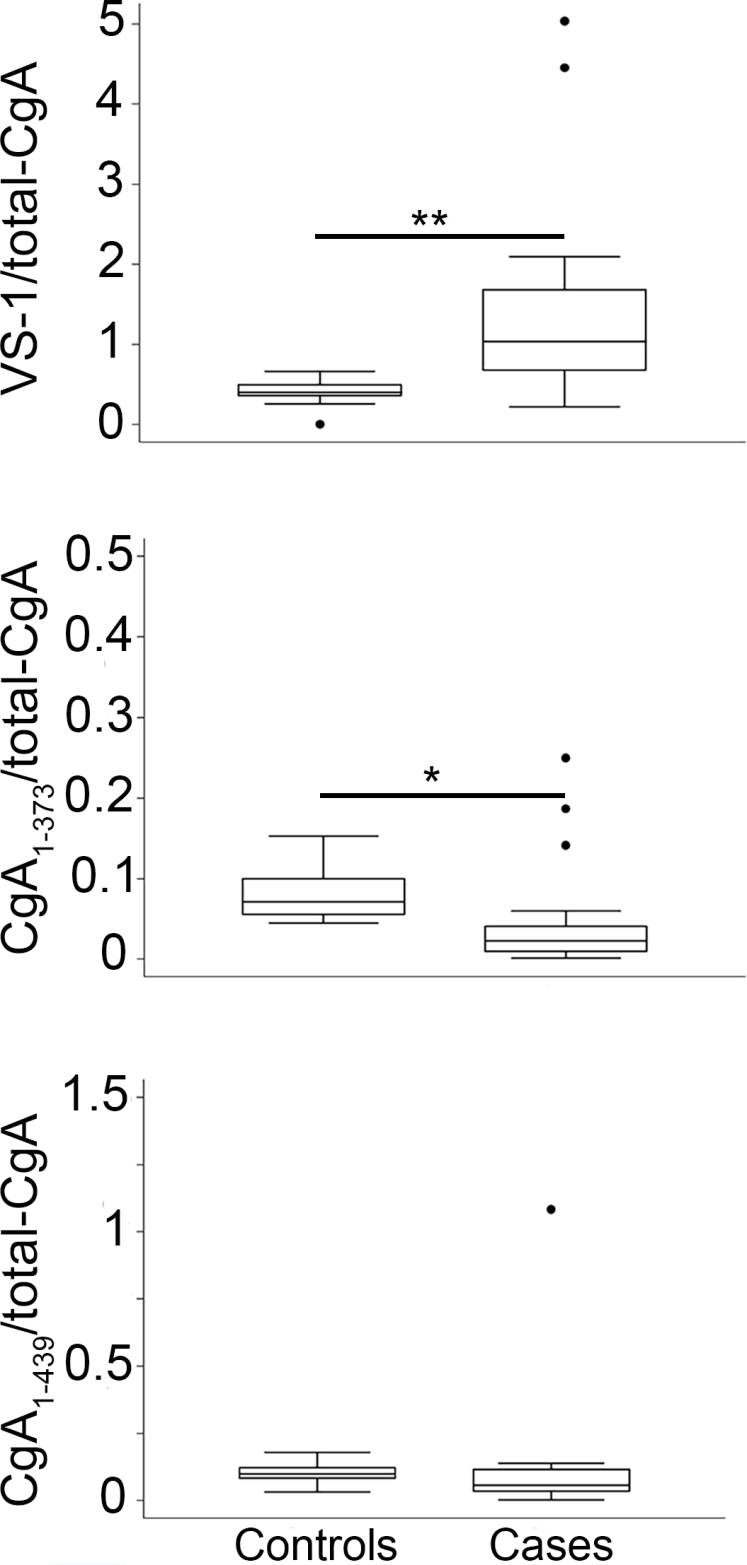
Levels of VS-1, CgA_1-373_ and CgA_1-439_ relative to the total-CgA in the plasma of patients with ileal and pancreatic NENs (cases) and healthy subjects (controls). Box-plots with median (middle line), 75^th^ percentile (top line) and 25^th^ percentile (bottom line). The top and bottom whiskers represent the upper and lower adjacent values, respectively. Values outside the whiskers are plotted individually (circles). Cases (n = 17); controls (n = 10). * (*p* < 0.05), **(*p* < 0.01), by analysis of covariance. As total-CgA does not include VS-1, VS-1/total-CgA ratio can be also greater than 1.

To evaluate whether the increased levels of circulating VS-1 in NENs was the result of increased CgA cleavage at the N-terminal region, or was simply related to increased CgA secretion, we analyzed the ratio of VS-1 and other fragments with total-CgA. Interestingly, we observed an increase of VS-1/total-CgA and a decrease of CgA_1-373_/total-CgA ratio in patients, compared to controls (*p* = 0.005 and *p* = 0.045, respectively) (Fig 3) (S2 Table). These data support the hypothesis that the proteolytic processing of CgA is altered in NENs favoring the production of the N-terminal fragment VS-1.

### The CgA processing is similar in ileal and pancreatic NENs

**Fig 4:**
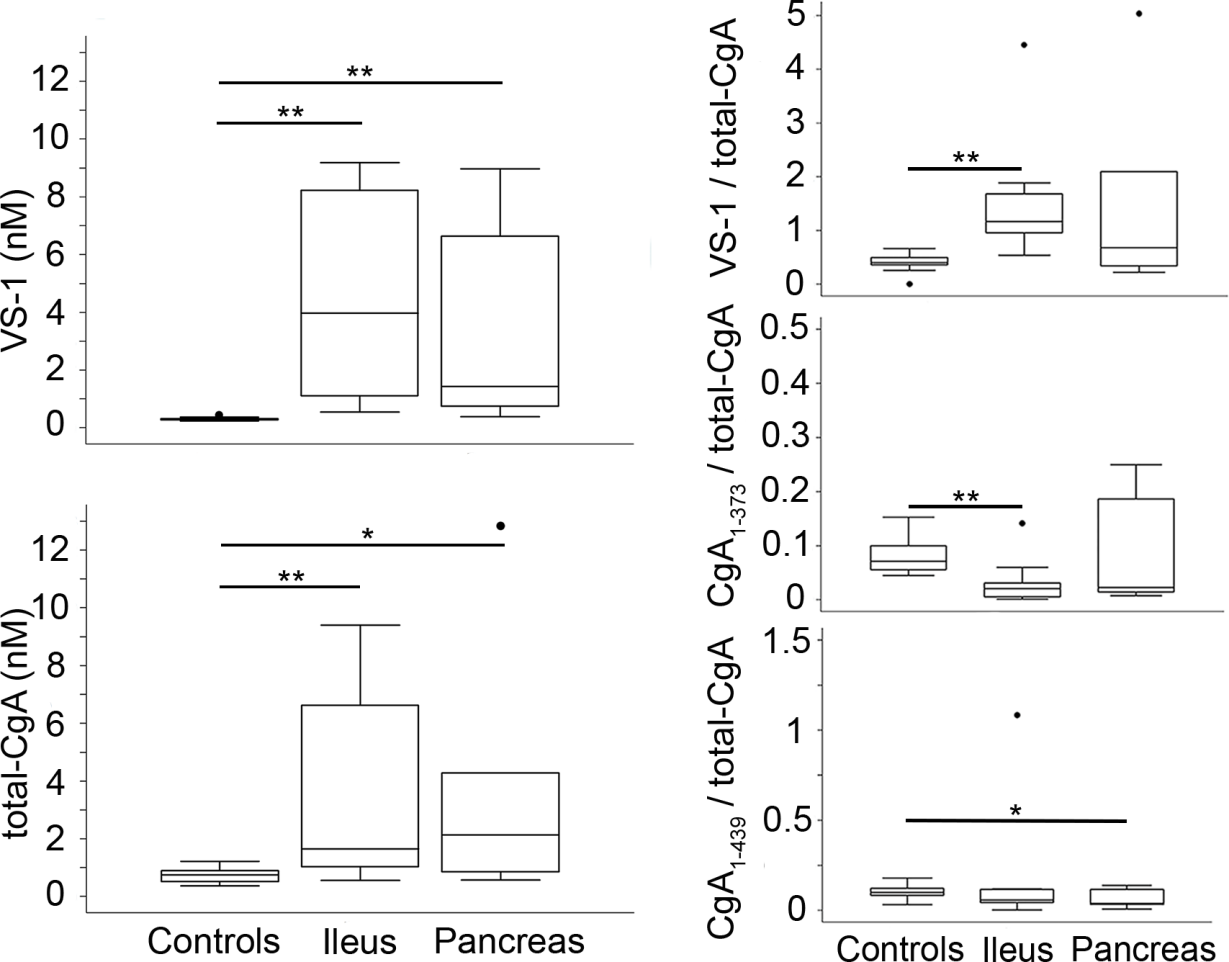
Plasma levels of VS-1 and total-CgA, VS-1/total-CgA, CgA_1-373_/total-CgA, and CgA_1-439_/total-CgA ratios (relative levels) in healthy subjects and patients with ileal and pancreatic NENs (case-control analysis by site). Box-plots with median (middle line), 75^th^ percentile (top line) and 25^th^ percentile (bottom line). The top and bottom whiskers represent the upper and lower adjacent values, respectively. Values outside the whiskers are plotted individually (circles). Ileal NENs (n = 10); pancreatic NENs (n = 7); controls (n = 10). * (*p* < 0.05), **(*p* < 0.01), by analysis of covariance.

The importance of tumor location on CgA production and cleavage was then investigated. Median total-CgA and VS-1 were significantly higher both in ileal and pancreatic cases compared to controls [ileal vs control, total-CgA: 1.655 nM (1.010–6.624) vs 0.752 nM (0.522–0.899), *p* = 0.009; VS-1: 3.966 nM (1.113–8.225) vs 0.288 nM (0.263–0.315), *p*<0.001] [pancreatic vs control, total-CgA: 2.129 nM (0.845–4.277) vs 0.753 nM (0.522–0.899), *p* = 0.014; VS-1: 1.433 nM (0.751–6.633) vs 0.288 nM (0.263–0.315), *p*<0.001] (Fig 4). VS-1/total-CgA was significantly increased in ileal but not in pancreatic NENs (*p*<0.001; *p* = 0.057, respectively). CgA_1-373_/total-CgA and CgA_1-439_/total-CgA were significantly lower in ileal and in pancreatic NENs, respectively (*p* = 0.007 and *p* = 0.037) (Fig 4). Furthermore, no differences in total-CgA, CgA_1-439_, CgA_1-373_, VS-1, CgA_1-439_/total-CgA, CgA_1-373_/total-CgA, VS-1/total-CgA, CgA_1-373_/CgA_1-439_ and CgA_1-373_/CgA_1-76_ emerged in the two groups (Fig 4, S2 Table). These results suggest that the proteolytic processing of CgA at the N-terminal region is increased in both ileal and pancreatic NENs when compared with healthy subjects.

### Correlation and ROC curve analysis of total-CgA and VS-1

**Fig 5:**
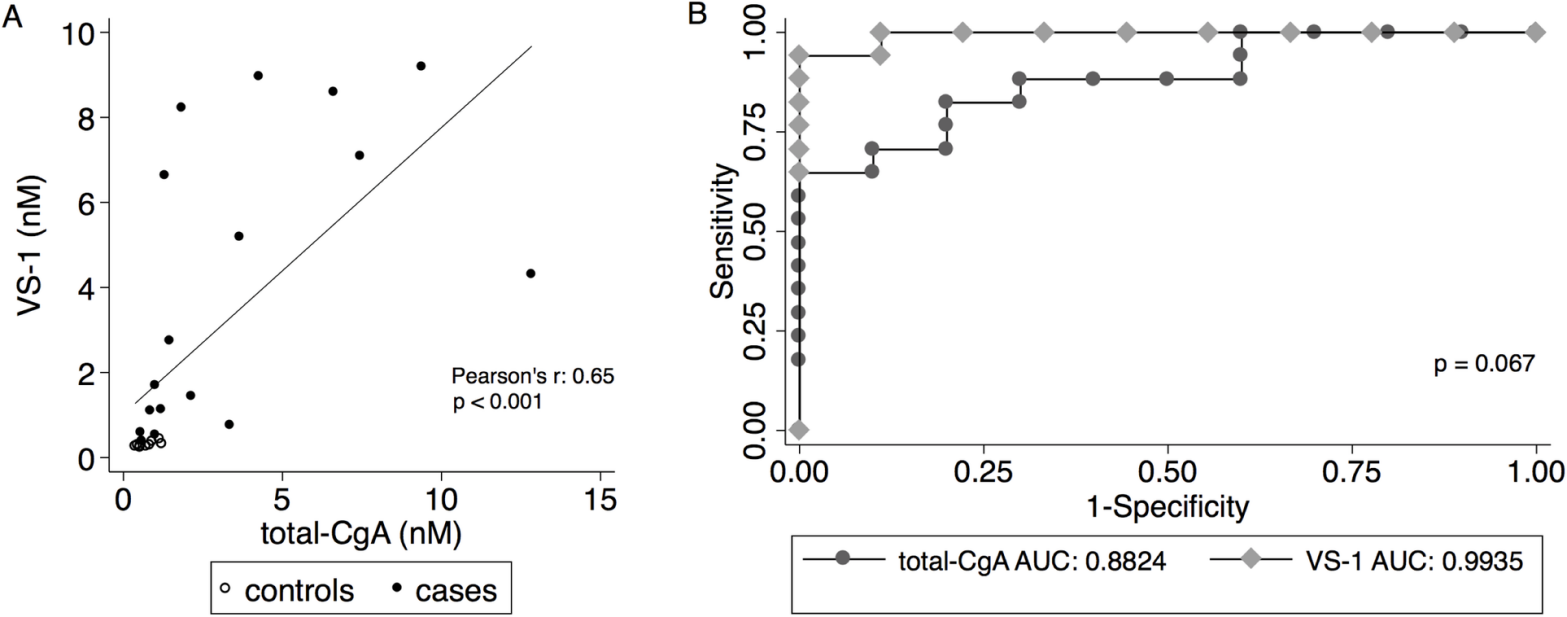
Correlation and receiver operating characteristic (ROC) curve of VS-1 and total-CgA. A) Correlation between the plasma levels of VS-1 and total-CgA in patients with NENs (solid circles) and healthy subjects (hollow circles). B) ROC curve of VS-1 and total-CgA.

To compare the capability of total-CgA and VS-1 to discriminate between patients and normal subjects we performed correlation and receiver operating characteristic (ROC) analysis. We observed a significant positive correlation between total-CgA and VS-1 (r = 0.65, *p*<0.001; Fig 5A). ROC analysis showed that the global performance (i.e. area under the curve) of VS-1 was 0.9935, whereas that of total-CgA was 0.8824 (*p* = 0.067; Fig 5B). The best cut-off for VS-1 was 0.442 nM, which correctly classified 96.1% of our patients, with a sensitivity of 94.1% and a specificity of 100%. The best cut-off for total-CgA was 0.899 nM, which correctly classified 81.5% of patients, with a sensitivity of 82.3% and a specificity of 80%. Notably, some patients had low levels of total-CgA but high levels of VS-1, whereas all healthy subjects had low levels of circulating VS-1 (Fig 5A and 5B). These results, overall, suggest that plasma VS-1 might represent an accurate biomarker for NENs comparable or possibly better than CgA.

### Somatostatin analogues affect both CgA and VS-1

**Fig 6:**
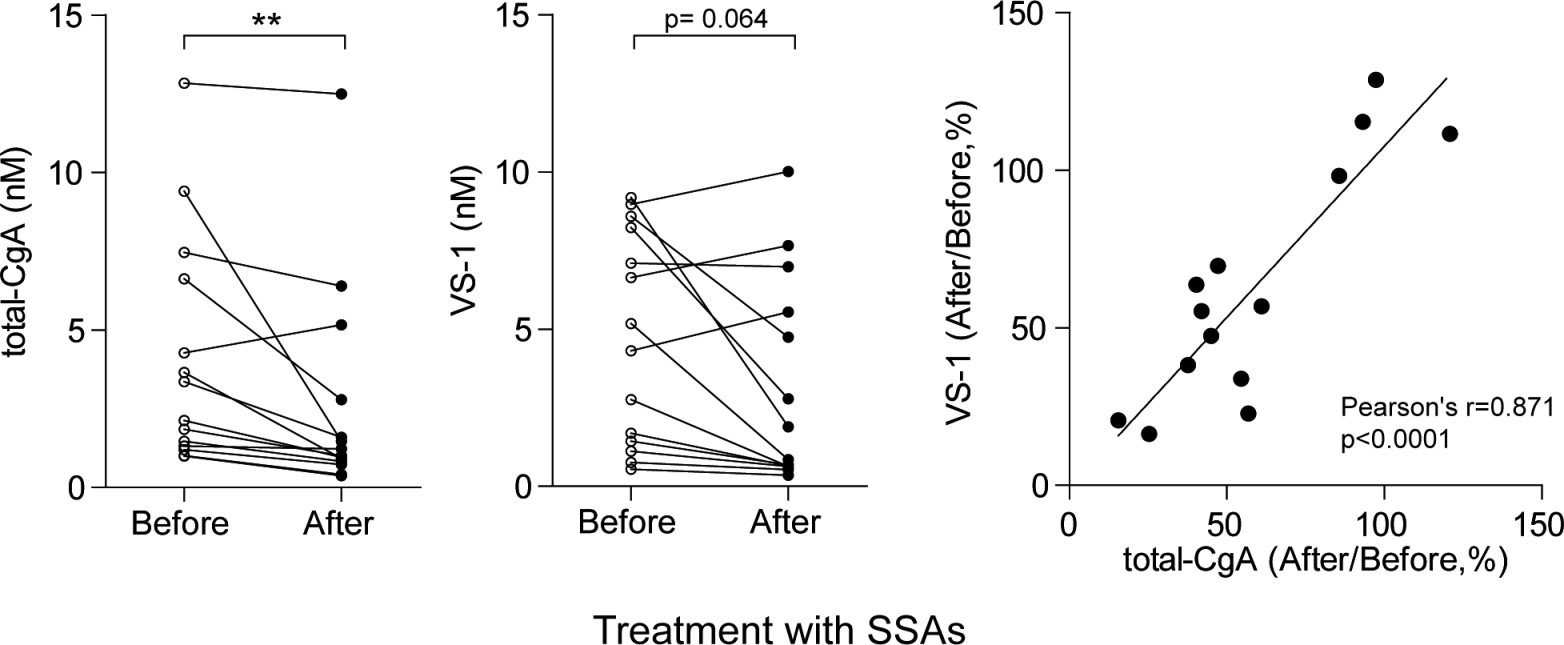
Effect of therapy with somatostatin analogues (SSAs) on the plasma levels of total-CgA and VS-1 in patients with ileal and pancreatic NENs. (A, B) Plasma levels of total-CgA and VS-1 in NENs cases (ileal plus pancreatic NENs) before and after the administration of octreotide LAR. ** (*p* < 0.01), by Wilcoxon signed-rank test. (C) Correlation between the decrease (%) of the plasma levels of VS-1 and total-CgA after therapy.

To provide further evidence that VS-1 can be exploited as a marker for pancreatic and ileal NENs follow-up, we then analyzed the effect of therapy with SSAs on VS-1 and total-CgA plasma levels in 14 patients untreated with PPIs. This treatment significantly reduced total-CgA (p<0.01). Furthermore, a not statistically significant (p = 0.064) decrease of VS-1 was also observed (Fig 6A and 6B). No change was observed for CgA_1-439_ or CgA_1-373_ (S3 Table). Interestingly, patients showing reduced levels of CgA also showed reduced levels of VS-1, as indicated by a good correlation between the % decrease of the plasma levels of both analytes after therapy (r = 0.871, p<0.0001; Fig 6C). Accordingly, no significant change of VS-1/total-CgA after therapy was observed (S3 Table). Moreover, no difference emerged between ileal and pancreatic NENs.

These data suggest that VS-1, like CgA, responds to therapy with SSAs.

### Proton-pump inhibitors increase the plasma levels of total-CgA but not of VS-1

**Fig 7:**
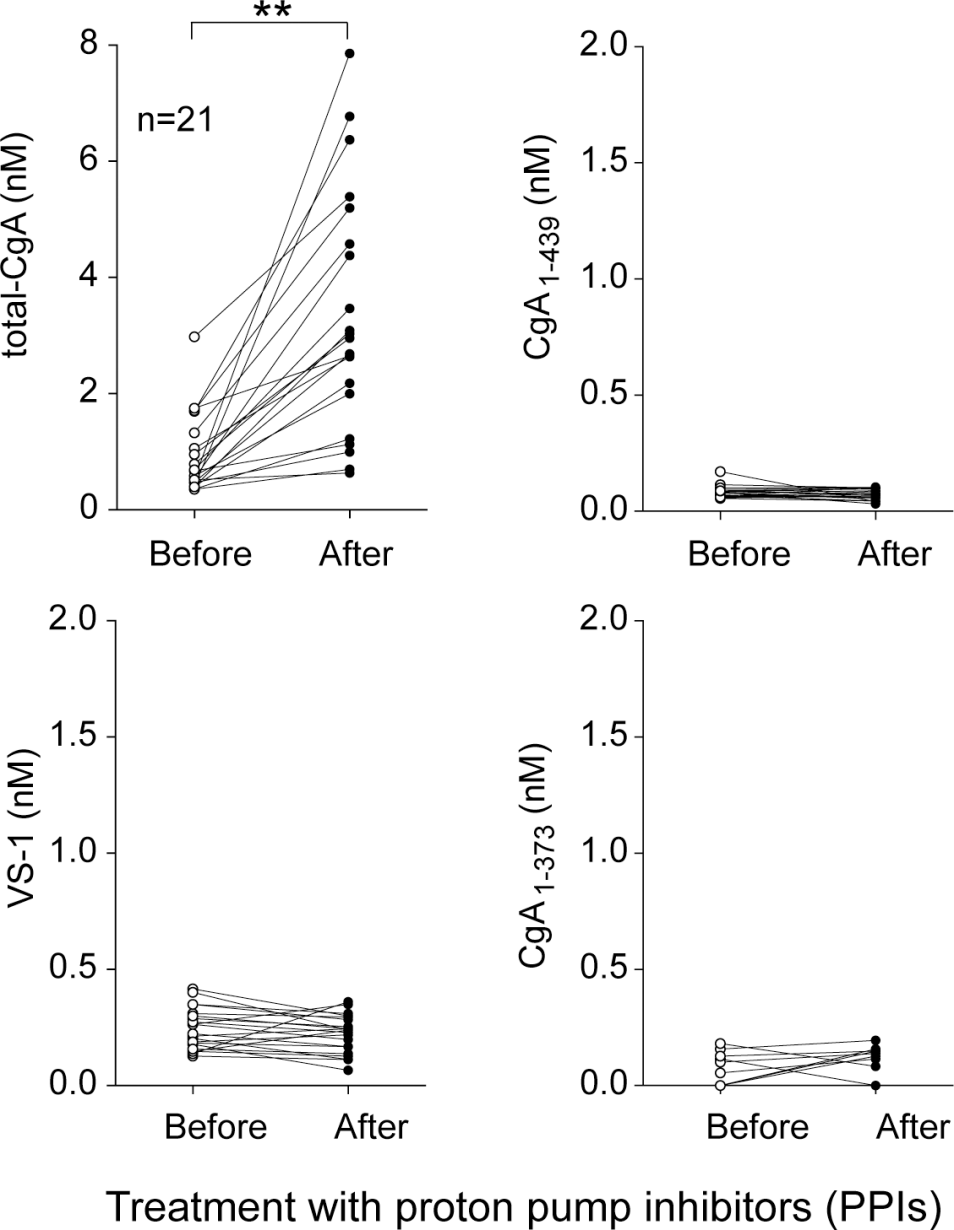
Effect of proton pump inhibitors on the plasma levels of total-CgA, VS-1, CgA_1-439_ and CgA_1-373_. **(*p* < 0.01), by two-tailed paired t-test.

To assess whether PPIs can differentially induce CgA and VS-1 in circulation, we administered pantoprazole (40 mg/day) to 21 healthy volunteers for 14 consecutive days. This treatment caused a marked increase in circulating total-CgA from 0.583 nM (0.466–1.058) to 2.958 nM (2.001–4.576) [median (25^th^-75^th^ percentiles), *p*<0.0001]. In contrast, no change in the levels of VS-1 was observed [mean (SD): from 0.244 nM (0.088) to 0.222 nM (0.081), *p* = 0.4] (Fig 7). These data suggest that PPIs can increase the circulating levels of CgA, in line with current literature, but not those of VS-1, suggesting that VS-1 is a biomarker more accurate and tumor-specific than CgA.

## Discussion

This study shows that plasma VS-1 is significantly increased in patients with pancreatic or ileal NENs compared to healthy subjects (5-fold and 14-fold, respectively). The observation that VS-1, total-CgA, and VS-1/total-CgA ratio (i.e. absolute and relative values of VS-1) were higher in NENs patients suggests that the increase of VS-1 plasma levels reflects an increased production of CgA as well as an increased cleavage at its N-terminal region. No significant difference was observed in patients with ileal or pancreatic NENs, suggesting that VS-1 cleavage occurs in both types of tumor tissues. Remarkably, the results also show that circulating VS-1, unlike total-CgA, did not increase after PPI administration in healthy volunteers. These data suggest that VS-1 represents a novel biomarker for ileal and pancreatic NENs. No significant difference was observed with CgA_1-439_ and CgA_1-373_.

It is well-known that the use of CgA as a NENs biomarker has several limitations. Firstly, many other pathologies, such as heart failure, renal failure, hypertension and various inflammatory diseases, can lead to an increase in its concentration [16]. Secondly, circulating CgA is increased after administration of PPIs, as a consequence to the fact that these drugs induce CgA-positive enterochromaffin-like cell hyperplasia [14, 15]. Thirdly, CgA is a highly heterogeneous analyte made by complex mixtures of full-length protein and fragments with different post-translational modifications [3]. Considering that most commercially available tests used for CgA quantification can detect full-length CgA and fragments (like our total-CgA-ELISA), these assays are difficult to standardize and can produce different results depending on antibody epitopes [23].

The observation that VS-1 is increased in NENs patients, but, unlike CgA, not in subjects treated with PPIs, and the notion that VS-1 is a well-defined analyte with no other post-translational modifications (thus easier to standardize than CgA), suggest that this fragment might represent an alternative or complementary biomarker for NENs diagnosis and/or follow up.

Interestingly, some patients with total-CgA close to normal values had abnormal levels of VS-1. Furthermore, the results of ROC analysis showed that VS-1 is as accurate, or even more, than CgA in discriminating patients and controls. These data, overall, suggest that this fragment might be more sensitive and specific than CgA as a NENs biomarker. Further studies on a larger sample of patients are needed to assess this hypothesis. Furthermore, other studies are necessary to assess whether comorbidities known to increase CgA levels can also increase the levels of circulating VS-1 or not. Interestingly, increased circulating levels of VS-1 have been observed so far in critically ill patients [34] and in patients with Takayasu arteritis [35], indicating that abnormal levels of VS-1 are not limited to NENs. However, it is important to note that, in critically ill patients, the VS-1 median value was 1.4-fold higher than that of healthy controls [34], an increase that is much lower than the 9.5-fold increase observed in our cohort of patients with NENs.

The observation that PPIs increase total-CgA levels, but not VS-1 levels, may suggest that enterochromaffin-like cells, which are thought to be the main source of PPI-induced CgA, do not produce the protease(s) responsible for CgA cleavage at its N-terminal domain. In contrast, ileal and pancreatic NENs tissues, which are expected to be the main source of the increased circulating CgA in patients, likely produce these proteases, leading to intra- and/or extra-cellular processing of CgA at its N-terminal domain, thereby increasing VS-1 production.

The fact that VS-1 and total-CgA are increased in patients with NENs raises the question as to whether these polypeptides are simply epiphenomena of the disease or they are somehow involved in the regulation of the tumor biology. Interestingly, previous studies have shown different expression of CgA in primary and metastatic cell lines [36]. Different levels of VS-1 and other CgA fragments have been also observed by immuno-histochemical and biochemical analysis of NENs from different sites [37–39]. Although VS-1 and CgA have been implicated as possible players in the regulation of angiogenesis and cell proliferation [20, 36, 40, 41], it is difficult to speculate whether the enhanced proteolytic processing of CgA in patients is a favorable or detrimental process, considering that the presence of VS-1 likely implicates the presence of other fragments, not tested in the present study, which might contribute to regulate tumor growth in an unpredictable manner. Further assays have to be developed to detect other CgA-derived peptides and to evaluate their implications in tumor biology.

In conclusion, the results of the present study suggest that the plasma levels of VS-1 are markedly increased in patients with ileal and pancreatic NENs and that this polypeptide, not affected by PPI therapy, might represent a novel biochemical marker for these NENs, easier to standardize and more accurate than CgA. Studies with a larger cohort of patients are necessary to assess how comorbidities might interfere with proteolytic processing of CgA, to evaluate the accuracy of VS-1 as an alternative or complementary NENs biomarker, and to assess its prognostic value.

## Supporting information

S1 Table ELISA precision and detectability. (DOCX)

S2 Table Absolute and relative plasma levels of CgA and its fragments in healthy subjects (controls) and patients with ileal or pancreatic NENs (cases). (DOCX)

S3 Table Effect of therapy with somatostatin analogues on the plasma levels of CgA and its fragments (absolute and relative levels) in patients with ileal and pancreatic NENs. (DOCX)
