# Supplementary material for: Vasostatin-1: A novel circulating biomarker for ileal and pancreatic neuroendocrine neoplasms
Source: PLoS One. 2018 May 3;13(5):e0196858. doi: 10.1371/journal.pone.0196858 (PMC5933774; doi:10.1371/journal.pone.0196858)
Supplement: S1 Table — (DOCX) [file pone.0196858.s001.docx]

| **Supporting Table 1. ELISA precision and detectability.** | | | | | |
| --- | --- | --- | --- | --- | --- |
| *Assay* | *Sample dilution* | *CV(%) ^a)^* | | *LoD ^b)^* | *LoQ ^c)^* |
|  |  | *Intra-assay* | *inter-assay* | *(nM)* | *(nM)* |
| **total-CgA-ELISA**^d)^ | 1:3 | 5.35 | 13.55 | 0.008 | 0.030 |
| ***439-ELISA**^e)^ | 1:2 | 4.08 | 9.99 | 0.005 | 0.017 |
| ***373-ELISA**^f)^ | 1:2 | 11.20 | 17.84 | 0.040 | 0.149 |
| ***76-ELISA**^g)^ | 1:2 | 5.69 | 14.15 | 0.027 | 0.063 |

1. Coefficient of variation (%), as estimated by mesuring 2-3 plasma samples 5 times in duplicate, 50 µl/well.
2. Limit of detection, as estimated by measuring 12 times the “zero standard” solution and by interpolating the mean + 2 SD on the calibration curve.
3. Limit of quantification, as estimated by measuring 12 times the “zero standard” solution and by interpolating the mean + 4 SD on the calibration curve and multiplying by sample dilution.
4. This assay can detect CgA_1-439_ and fragments lacking the C-terminal region, but not VS-1 [19].
5. This assay can detect CgA_1-439_ but not CgA fragments [19].
6. This assay can detect CgA_1-373_ but not CgA_1-439_, or VS-1 [20].
7. This assay can detect CgA_1-76_ (VS-1), but not CgA_1-439_ or other larger fragments [19].
