# Supplementary material for: Vasostatin-1: A novel circulating biomarker for ileal and pancreatic neuroendocrine neoplasms
Source: PLoS One. 2018 May 3;13(5):e0196858. doi: 10.1371/journal.pone.0196858 (PMC5933774; doi:10.1371/journal.pone.0196858)
Supplement: S2 Table — (DOCX) [file pone.0196858.s002.docx]

| Supporting Table 2. Absolute and relative plasma levels of CgA and its fragments in healthy subjects (controls) and patients with ileal or pancreatic NENs (cases). | | | |
| --- | --- | --- | --- |
|  | **Case** | **Control** | **p-value*** |
| ***Case-control Analysis*** | median  (25^th^-75^th^ percentiles) | median  (25^th^-75^th^ percentiles) |  |
| **total-CgA** | 1.848  (1.010-4.276) | 0.752  (0.522-0.899) | *0.004* |
| **CgA_1-439_** | 0.066  (0.050-0.193) | 0.072  (0.062-0.095) | *0.162* |
| **CgA_1-373_** | 0.057  (0.014-0.142) | 0.057  (0.037-0.067) | *0.968* |
| **VS-1** | 2.757  (1.093-7.098) | 0.288  (0.263-0.315) | *<0.001* |
| **CgA_1-439_/total-CgA** | 0.056  (0.034-0.115) | 0.098  (0.083-0.122) | *0.103* |
| **CgA_1-373_/total-CgA** | 0.022  (0.009-0.040) | 0.071  (0.055-0.099) | *0.045* |
| **VS-1/total-CgA** | 1.036  (0.682-1.683) | 0.397  (0.360-0.498) | *0.005* |
| **CgA_1-373_/CgA_1-439_** | 0.584  (0.081-1.374) | 0.744  (0.578-1.139) | *0.438* |
| **CgA_1-373_/VS-1** | 0.023  (0.007-0.034) | 0.157  (0.149-0.210) | *0.001* |
| ***Ileal-control Analysis*** |  | | |
| **total-CgA** | 1.655  (1.010-6.624) | 0.752  (0.522-0.899) | *0.009* |
| **CgA_1-439_** | 0.102  (0.057-0.193) | 0.072  (0.062-0.095) | *0.086* |
| **CgA_1-373_** | 0.036  (0.011-0.142) | 0.057  (0.037-0.067) | *0.573* |
| **VS-1** | 3.966  (1.113-8.225) | 0.288  (0.263-0.315) | *<0.001* |
| **CgA_1-439_/total-CgA** | 0.056  (0.044-0.116) | 0.098  (0.083-0.122) | *0.328* |
| **CgA_1-373_/total-CgA** | 0.021  (0.005-0.031) | 0.071  (0.055-0.099) | *0.007* |
| **VS-1/total-CgA** | 1.166  (0.951-1.683) | 0.397  (0.360-0.498) | *<0.001* |
| **CgA_1-373_/CgA_1-439_** | 0.291  (0.069-1.144) | 0.744  (0.578-1.139) | *0.214* |
| **CgA_1-373_/VS-1** | 0.009  (0.006-0.027) | 0.157  (0.149-0.210) | *<0.001* |
| *Analysis of Covariance | | | |

| Supporting Table 2 continued. | | | |
| --- | --- | --- | --- |
|  | **Case** | **Control** | **p-value*** |
| ***Pancreas-control Analysis*** | median  (25^th^-75^th^ percentiles) | median  (25^th^-75^th^ percentiles) |  |
| **total-CgA** | 2.129  (0.845-4.277) | 0.752  (0.522-0.899) | *0.014* |
| **CgA_1-439_** | 0.066  (0.034-0.311) | 0.072  (0.062-0.095) | *0.716* |
| **CgA_1-373_** | 0.061  (0.040-0.157) | 0.057  (0.037-0.067) | *0.177* |
| **VS-1** | 1.433  (0.751-6.633) | 0.288  (0.263-0.315) | *<0.001* |
| **CgA_1-439_/total-CgA** | 0.039  (0.032-0.116) | 0.098  (0.083-0.122) | *0.037* |
| **CgA_1-373_/total-CgA** | 0.022  (0.014-0.186) | 0.071  (0.055-0.099) | *0.371* |
| **VS-1/total-CgA** | 0.682  (0.336-2.097) | 0.397  (0.360-0.498) | *0.057* |
| **CgA_1-373_/CgA_1-439_** | 1.184  (0.160-2.161) | 0.744  (0.578-1.139) | *0.544* |
| **CgA_1-373_/VS-1** | 0.034  (0.008-0.144) | 0.157  (0.149-0.210) | *0.020* |
|  |  |  |  |
|  | **Ileus** | **Pancreas** | **p-value*** |
| ***Site Analysis*** |  | | |
| **total-CgA** | 1.655  (1.010-6.624) | 2.129  (0.845–4.277) | *1.0* |
| **CgA_1-439_** | 0.102  (0.057-0.193) | 0.066  (0.034–0.311) | *0.696* |
| **CgA_1-373_** | 0.036  (0.011-0.142) | 0.061  (0.040–0.157) | *0.261* |
| **VS-1** | 3.966  (1.113-8.225) | 1.433  (0.751–6.633) | *0.435* |
| **CgA_1-439_/total-CgA** | 0.056  (0.044-0.116) | 0.039  (0.032–0.116) | *0.558* |
| **CgA_1-373_/total-CgA** | 0.021  (0.005-0.031) | 0.022  (0.014–0.186) | *0.329* |
| **VS-1/total-CgA** | 1.166  (0.951-1.683) | 0.682  (0.336–2.097) | *0.379* |
| **CgA_1-373_/CgA_1-439_** | 0.291  (0.069-1.144) | 1.184  (0.160–2.161) | *0.143* |
| **CgA_1-373_/VS-1** | 0.009  (0.006-0.027) | 0.034  (0.008–0.034) | *0.097* |
| *Analysis of Covariance | | | |
