# Supplementary material for: Vasostatin-1: A novel circulating biomarker for ileal and pancreatic neuroendocrine neoplasms
Source: PLoS One. 2018 May 3;13(5):e0196858. doi: 10.1371/journal.pone.0196858 (PMC5933774; doi:10.1371/journal.pone.0196858)
Supplement: S3 Table — (DOCX) [file pone.0196858.s003.docx]

| Supporting Table 3. Effect of therapy with somatostatin analogues on the plasma levels of CgA and its fragments (absolute and relative levels) in patients with ileal and pancreatic NENs. | | |
| --- | --- | --- |
|  | **Difference in plasma levels after SSAs** | **p-value*** |
| ***All cases*** | median  (25^th^-75^th^ percentiles) |  |
| **total-CgA** | -0.736  (-1.777 – -0.468) | *0.005* |
| **CgA_1-439_** | -0.015  (-0.033 – 0.009) | *0.177* |
| **CgA_1-373_** | 0.001  (-0.059 – 0.017) | *0.379* |
| **VS-1** | -0.617  (-3.841 – -0.121) | *0.064* |
| **CgA_1-439_/total-CgA** | 0.014  (0.002 – 0.055) | *0.022* |
| **CgA_1-373_/total-CgA** | 0.025  (-0.002 – 0.042) | *0.048* |
| **VS-1/total-CgA** | 0.071  (-0.159 – 0.311) | *0.730* |
| **CgA_1-373_/CgA_1-439_** | 0.075  (-0.704 – 0.428) | *0.826* |
| **CgA_1-373_/VS-1** | 0.011  (-0.003 – 0.024) | *0.158* |
| ***Ileus*** |  |  |
| **total-CgA** | -0.839  (-2.719 – -0.265) | *0.008* |
| **CgA_1-439_** | -0.016  (-0.031 – -0.008) | *0.109* |
| **CgA_1-373_** | 0.002  (-0.059 – 0.008) | *0.374* |
| **VS-1** | -2.127  (-4.333 – -0.481) | *0.008* |
| **CgA_1-439_/total-CgA** | 0.028  (0.000 – 0.096) | *0.173* |
| **CgA_1-373_/total-CgA** | 0.029  (0.000 – 0.046) | *0.051* |
| **VS-1/total-CgA** | 0.027  (-0.511 – 0.311) | *0.678* |
| **CgA_1-373_/CgA_1-439_** | 0.799  (-0.704 – 0.428) | *0.953* |
| **CgA_1-373_/VS-1** | 0.018  (0.001 – 0.024) | *0.086* |
| *Wilcoxon signed-rank test | | |

| Supporting Table 3 continued. | | |
| --- | --- | --- |
|  | **Difference in plasma levels after SSAs** | **p-value*** |
| ***Pancreas*** | median  (25^th^-75^th^ percentiles) |  |
| **total-CgA** | -0.344  (-1.170 – -0.091) | *0.225* |
| **CgA_1-439_** | 0.006  (-0.033 – 0.014) | *0.893* |
| **CgA_1-373_** | 0.000  (-0.023 – 0.044) | *1* |
| **VS-1** | 1.023  (-0.228 – 1.043) | *0.225* |
| **CgA_1-439_/total-CgA** | 0.008  (0.005 – 0.013) | *0.043* |
| **CgA_1-373_/total-CgA** | -0.001  (-0.002 – 0.039) | *0.686* |
| **VS-1/total-CgA** | 0.106  (0.036 – 0.108) | *0.345* |
| **CgA_1-373_/CgA_1-439_** | -0.067  (-0.113 – 0.351) | *0.893* |
| **CgA_1-373_/VS-1** | -0.001  (-0.003 – 0.005) | *0.893* |
| *Wilcoxon signed-rank test | | |
